# Prevention of taxane chemotherapy-induced nail changes and peripheral neuropathy by application of extremity cooling: a prospective single-centre study with intrapatient comparison

**DOI:** 10.1007/s00520-024-08737-3

**Published:** 2024-07-27

**Authors:** Kristen Johnson, Barbara Stoffel, Michael Schwitter, Stefanie Hayoz, Alfonso Rojas Mora, Angela Fischer Maranta, Tämer El Saadany, Ursula Hasler, Roger von Moos, Annalea Patzen, Michael Mark, Gillian Roberts, Richard Cathomas

**Affiliations:** 1 Division of Oncology/Hematology, Kantonsspital Graubünden, Chur, Switzerland; 2 Department of Internal Medicine, Kantonsspital Graubünden, Chur, Switzerland; 3 SAKK Competence Center, Bern, Switzerland; 4 University of Zürich, Zurich, Switzerland

**Keywords:** Chemotherapy-induced polyneuropathy (CIPN), Nail changes, Paclitaxel, Docetaxel, Hilotherapy

## Abstract

**Purpose:**

Common side effects of taxane chemotherapy are nail toxicity and peripheral neuropathy (CIPN) causing severe impact on the quality of life. Different methods of cryotherapy to prevent these side effects have been tested. We investigated the use of machine-controlled cooling of hands and feet to reduce nail toxicity and CIPN in patients receiving taxane chemotherapy.

**Methods:**

Patients receiving Docetaxel (planned dose $\ge$ 300 mg/m^2^) or Paclitaxel (planned dose $\ge$ 720 mg/m^2^ $-$) in the adjuvant or palliative setting of different cancers were included. The dominant hand and foot were cooled to approximately 10 °C using the Hilotherapy machine. The contralateral hand and foot were used as intrapatient comparison. The primary endpoint was the occurrence of any CIPN due to paclitaxel or nail toxicity due to Docetaxel. Both the intention to treat population (ITT) and the per protocol population (PPP) were analyzed.

**Results:**

A total of 69 patients, 21 treated with Docetaxel and 48 with Paclitaxel, were included at our centre between 08/2020 and 08/2022. Nail toxicity due to Docetaxel was overall not significantly improved by cooling in the ITT or PPP but a significant benefit across visits was found for the ITT. CIPN due to Paclitaxel was numerically better in the ITT and significantly better in the PPP. A significant benefit of cooling on CIPN occurrence across visits was found for the ITT and the PPP. Cooling was very well tolerated.

**Conclusion:**

Cooling of hands and feet has a clinically meaningful impact on reducing occurrence of CIPN and nail toxicity on treatment with taxanes. Effects are more significant over time and are dose dependent.

Trial registration number.

2020–00381.

Date of registration.

24th February 2020.

**Supplementary Information:**

The online version contains supplementary material available at 10.1007/s00520-024-08737-3.

## Introduction

Taxanes, including Paclitaxel and Docetaxel, are microtubule inhibitors discovered in the 1960s [1, 2]. These chemotherapeutic agents have a specific adverse effect profile including nail toxicity and peripheral neuropathy [3–5].

Nail toxicity is seen in up to 51% of patients with the use of Docetaxel [1, 3, 6–8]. Many authors have described significant underreporting and some have reported nail changes in 85% of patients [1, 7, 9]. In comparison, the incidence of nail toxicity caused by Paclitaxel is much lower and in the range of 25% [6]. Nail toxicity varies from cosmetic changes such as discoloration, Beau’s lines, onychomadesis, melanonychia and splinter haemorrhage up to more severe effects such as painful paronychia, onycholysis and subungual hematoma [10].

Chemotherapy-induced polyneuropathy (CIPN) is another common adverse effect caused by many cytotoxic chemotherapy agents [4, 11, 12]. CIPN induced by taxanes, especially Paclitaxel, is very common and has been extensively described [13, 14]. Incidences of Paclitaxel-induced CIPN are high, reaching up to 93%, with strong indication of dose-dependence [11, 15–18]. Docetaxel-induced CIPN is less common but is also well known and mainly dose dependent and with a variety of incidences [15, 18]. CIPN is mainly of the sensory type and varies from paraesthesia, numbness and tingling to severe cases including pain, as well as deficiencies in walking ability and motor skills, resulting in a reduction in daily functionality [11, 13, 18–20].

Both nail changes and CIPN have severe impacts on the quality of life of the patients undergoing chemotherapy [1, 4, 5, 7, 12, 15, 18–20]. Symptoms, especially mild to moderate CIPN, can persist long after cessation of chemotherapy [4, 5, 11, 12, 21]. These adverse effects can lead to discontinuation, dose reduction or switch of chemotherapy, potentially reducing the effectiveness (for nail toxicity [1, 10, 22, 23], for CIPN [5, 12, 13, 15, 18, 21]).

Different ways to prevent nail changes and CIPN caused by taxane have been investigated using the understanding that vasoconstriction can prevent the chemotherapy reaching the vessels of finger and toe tips. In particular, there have been several studies testing different methods of cryotherapy [3, 15, 17]. The POLAR trial conducted in 2022 showed a high efficacy in preventing ≥ grade 2 sensory CIPN using frozen gloves as well as compression therapy with surgical gloves [24].

A recent development is the application of extremity cooling (called Hilotherapy) using a Hilotherm ChemoCare machine, which contains special cuffs to cool the hand and foot to 10–12 °C. Two single arm studies have used the Hilotherapy machine and describe reduction of CIPN under treatment with Paclitaxel. Both studies applied the Hilotherapy on all extremities, therefore not having a direct comparator [25, 26]. Both studies conclude that Hilotherapy could represent a promising tool to reduce this sort of toxicity and merits further investigation.

Our trial aims to assess the real impact of Hilotherapy on the reduction of CIPN and nail toxicity in patients undergoing taxane chemotherapy by using intrapatient comparison.

## Methods

### Study design and objectives

We designed a prospective single-centre, open-label phase II trial. Patients receiving Paclitaxel (planned per protocol dose ≥ 720 mg/m^2^) or Docetaxel (planned per protocol dose ≥ 300 mg/m^2^) in an adjuvant or palliative setting were eligible for participation. Different dosing schedules of Paclitaxel or Docetaxel were allowed (weekly, every 2 weeks (q2w) or every 3 weeks (q3w)). Patients were excluded in cases of known hypersensitivity to Docetaxel or Paclitaxel or if they had pre-existing peripheral neuropathy > grade 1 or nail changes > grade 1.

All patients received cooling with the Hilotherapy machine of their dominant hand and foot of the same side to approximately 10–12 °C. Cooling started 30 min prior to chemotherapy and continued until 30 min after the end of the chemotherapy infusion. The hand and foot of the contralateral side was used as an intrapatient comparison. In case of occurrence of nail toxicity or CIPN, cooling of the control side at patient wish was allowed.

The primary objective was to investigate the beneficial preventive effects of the Hilotherapy machine to reduce nail toxicity due to Docetaxel and CIPN due to Paclitaxel. Secondary objectives included the occurrence of any nail toxicity under Paclitaxel treatment, as well as the occurrence of any CIPN under Docetaxel treatment regardless of cooling. Furthermore, the tolerability of the Hilotherapy was assessed.

The study protocol was approved by the local independent review board and conducted according to provisions of the Declaration of Helsinki and Good Clinical Practice Guidelines of the International Conference on Harmonization. All patients provided written informed consent.

### The Hilotherapy machine

The medical device used was the Hilotherm Chemo-Care-machine (Hilotherm GmbH in 88,260 Argenbühl-Eisenharz, Germany). It provides a controlled cooling of hands and feet to a target temperature of 10–12 °C. The Hilotherm ChemoCare machine contains special cuffs for the hand and foot. A cooling agent (distilled water) flows through several pipes located in the cuffs. Sensors are continuously monitoring the temperature of the cooling agent and therefore can cool the extremities to a specific desired temperature.

### Assessments

The Hilotherapy was installed at every cycle visit with the same procedure. Nail toxicity and CIPN were assessed in all patients at baseline and subsequently as follows: for weekly chemotherapy, on the 4th, 8th and 12th administration, corresponding to days 21, 49 and 77 (± 7 days); for q2w chemotherapy, on every second cycle, corresponding to days 28, 56, 84 and 110 (± 7 days); and for q3w chemotherapy, at the start of every cycle, corresponding to days 21, 42, 63, 84, 105 and 126 (± 7 days). End of treatment assessments occurred at 14–28 and 42–58 days after the last chemotherapy administration. Documentation of nail changes was done by specialist oncology nurses using a prespecified form (see online resource 1). A photograph was taken in case of any detected changes. Using this information, nail changes were graded by CTCAE version 5.0.

To assess CIPN incidence, patients filled out the PNQ questionnaire (Patient Neurotoxicity Questionnaire) containing questions about their sensory functions (i.e. numbness) as well as motor functions (i.e. weakness) (online resource 2). Depending on the severity of numbness or weakness, the patients had to answer how activities of daily life were restricted. Based on these questionnaires, specialist oncology nurses graded the CIPN for each extremity using the CTCAE version 5.0 (online resource 3). The CTCAE v.5 grading defines CIPN grade I as being asymptomatic with diagnostic observations only or having mild symptoms (e.g. paresthesia or loss of tendon reflexes), grade II as having moderate symptoms limiting instrumental activities of daily life, grade III as having severe symptoms limiting self-care and grade IV as having life-threatening symptoms requiring urgent intervention. The highest grade in each patient on cooled or non-cooled extremities was used for reporting. Collected data were entered into a previously designed database.

A summary table listing the schedule of assessments is added in the supplementary information (online resource 4).

### Statistical analysis

The primary endpoint was the occurrence of any CIPN in either hands or feet at any time during treatment (and up to 56 days after end of treatment) for the patients receiving Paclitaxel, and occurrence of nail toxicity in either hands or feet at any time during treatment (and up to 56 days after end of treatment) for the patients receiving Docetaxel.

Using a two-sided McNemar’s test for paired observations with a significance level of 5% and 90% power, a total of 24 patients per cohort was needed to show clinically relevant differences in the occurrence of toxicities between cooled and control extremities. To account for dropouts, 30 patients per cohort were planned to be recruited. The control and cooled extremities were compared using a logistic regression model, from which the odds ratio with its corresponding 95% confidence interval was reported.

Additionally, the treatment effect on the occurrence of toxicities over time was tested using a logistic mixed regression model. The occurrence of toxicities was used as response variable. The explanatory variables were visit, treatment (cooling vs. no cooling) and their interaction, while the patient was used as a random intercept with the treatment nested within patient. An analysis of deviance with type II sums of errors was computed to assess the significance of the fixed terms.

Categorical variables were summarized using frequency and percentage. Continuous variables were summarized using median and range.

All analyses were conducted for both the intention to treat population (ITT) and the per protocol population (PPP). Additionally, we conducted analyses considering adverse events occurring only in hands and only in feet.

All analyses were conducted in SAS 9.4 (SAS Institute Inc.) and R 4.2.2. Results were considered significant using an *α* = 0.05.

## Results

### Patient population

In total, 70 patients were accrued to the study between 8/2020 and 8/2022. One of the patients did not fulfil the eligibility criteria and two had missing treatment information, resulting in 67 patients evaluable for the primary endpoint. Sixty-nine were evaluable for the safety population.

A total of 21 patients were treated with Docetaxel (intention to treat population, ITT) of which 9 received at least 300 mg/m^2^ (per protocol population, PPP). The median total dose of Docetaxel in the ITT was 294 mg/m^2^ (min. 135 mg/m^2^, max. 455 mg/m^2^) and 402 mg/m^2^ (min. 380 mg/m^2^, max. 455 mg/m^2^) in the PPP. Docetaxel dose was reduced in five patients (23.8%) and interrupted in another five patients (23.8%).

A total of 46 patients were treated with Paclitaxel (ITT) of which 34 received at least 720 mg/m^2^ (PPP). The median total dose of Paclitaxel in the ITT was 813 mg/m^2^ (min. 72 mg/m^2^, max. 1326 mg/m^2^) and 891 mg/m^2^ (min. 741 mg/m^2^, max. 1326 mg/m^2^) in the PPP. The 12 patients, who did not receive a minimum dose of 720 mg/m^2^ and therefore were not included in the PPP, received a median dose of 422 mg/m^2^.

Paclitaxel dose was reduced in 22 patients (45.8%) and interrupted in 15 patients (31.3%). Reasons for reductions in dose were due to various reasons, including the deterioration of general condition, myelosuppression and refractory nausea. In three patients, dose was reduced due to progressing CIPN higher than grade II (online resource 5).

Baseline characteristics for ITT and PPP for each cohort are shown in Table 1.

**Table 1:** (a) Baseline characteristics of the study population (ITT). (b) Baseline characteristics of the study population (PPP)

(a)		
**Baseline characteristics ITT**	**Docetaxel**, *N* = 21; *n* (%)^1^	**Paclitaxel**, *N* = 46; *n* (%)^1^
**Median age (range)**	66 (37 – 81)	57 (28 – 86)
**Sex**		
Female	9 (42.9%)	38 (82.6%)
Male	12 (57.1%)	8 (17.4%)
**Tumour type**		
Breast cancer	8 (38.1%)	29 (63.0%)
NSCLC^*^	3 (14.3%)	1 (2.2%)
Ovary	-	2 (4.3%)
Prostate cancer	10 (47.6%)	2 (4.3%)
SCLC^**^	-	2 (4.3%)
Other	-	10 (21.7%)
**Tumour stage**		
I	4 (19.0%)	4 (8.7%)
II	3 (14.3%)	12 (26.1%)
III	1 (4.8%)	8 (17.4%)
IV	8 (38.1%)	12 (26.1%)
Unknown	5 (23.8%)	10 (21.7%)
**Tumour status**		
Local	5 (23.8%)	21 (45.7%)
Locally advanced	2 (9.5%)	6 (13.0%)
Metastatic	14 (66.7%)	19 (41.3%)
**Relapsed**		
No	16 (76.2%)	42 (91.3%)
Yes	5 (23.8%)	4 (8.7%)
**Diabetes**		
No	19 (90.5%)	42 (91.3%)
Type II	2 (9.5%)	4 (8.7%)
**Cardiovascular disease**		
No	15 (71.4%)	36 (78.3%)
Yes	6 (28.6%)	10 (21.7%)
Neurological disease***		
No	20 (95.2%)	43 (93.5%)
Yes	1 (4.8%)	3 (6.5%)
**Smoking status**		
Yes	4 (19.0%)	11 (23.9%)
Former	5 (23.8%)	7 (15.2%)
No	10 (47.6%)	28 (60.9%)
Unknown	2 (9.5%)	-
**Excessive alcohol consumption**		
No	19 (90.5%)	44 (95.7%)
Yes	2 (9.5%)	2 (4.3%)
**(b)**		
**Baseline characteristics PPP**	**Docetaxel**, *N* = 9; *n* (%)^1^	**Paclitaxel**, *N* = 34; *n* (%)^1^
**Median age (range)**	72 (66 – 81)	53 (28 – 81)
**Sex**		
Female	-	32 (94.1%)
Male	9 (100.0%)	2 (5.9%)
**Tumour type**		
Breast cancer	-	26 (76.5%)
NSCLC^*^	-	-
Ovary	-	2 (5.9%)
Prostate cancer	9 (100.0%)	1 (2.9%)
SCLC^**^	-	1 (2.9%)
Other	-	4 (11.8%)
**Tumour stage**		
I	-	4 (11.8%)
II	1 (11.1%)	10 (29.4%)
III	1 (11.1%)	6 (17.6%)
IV	4 (44.4%)	6 (17.6%)
Unknown	3 (33.3%)	8 (23.5%)
**Tumour status**		
Local	-	18 (52.9%)
Locally advanced	1 (11.1%)	5 (14.7%)
Metastatic	8 (88.9%)	11 (32.4%)
**Relapsed**		
No	5 (55.6%)	32 (94.1%)
Yes	4 (44.4%)	2 (5.9%)
**Diabetes**		
No	8 (88.9%)	33 (97.1%)
Type II	1 (11.1%)	1 (2.9%)
**Cardiovascular disease**		
No	6 (66.7%)	29 (85.3%)
Yes	3 (33.3%)	5 (14.7%)
Neurological disease***		
No	9 (100.0%)	32 (94.1%)
Yes	-	2 (5.9%)
**Smoking status**		
Yes	1 (11.1%)	9 (26.5%)
Former	4 (44.4%)	5 (14.7%)
No	4 (44.4%)	20 (58.8%)
Unknown	-	-
**Excessive alcohol consumption**		
No	8 (88.9%)	33 (97.1%)
Yes	1 (11.1%)	1 (2.9%)

^1^Median (range); *n* (%)

^*^Non-small cell lung cancer

^**^Small cell lung cancer

***Including multiple sclerosis, epilepsy, Parkinson’s disease and stroke

### Nail toxicity with Docetaxel

Nineteen patients (90%) treated with Docetaxel in the ITT group and nine patients (100%) in the PPP experienced some pathological nail change across all visits. There were no significant differences in the occurrence of pathological nail changes between cooled (81.0%; 95% CI, 63.7–97.0%) vs. not cooled (85.7%; 95% CI, 58.1–94.6%) extremities (odds ratio 0.71; 95% CI, 0.14–3.64; McNemar’s *p*-value = 0.56).

Looking at hands and feet separately, the ITT population showed a significant difference in the occurrence of pathological nail changes between cooled (52.4%; 95% CI, 29.8–74.3%) vs. not cooled (71.4%; 95% CI, 47.8–88.7%) for the feet (OR 0.44; 95% CI, 0.12–1.58; McNemar’s *p*-value = 0.046). There were no significant differences in the hand analysis of the ITT population nor in the PPP overall (data not shown).

**Fig. 1:**
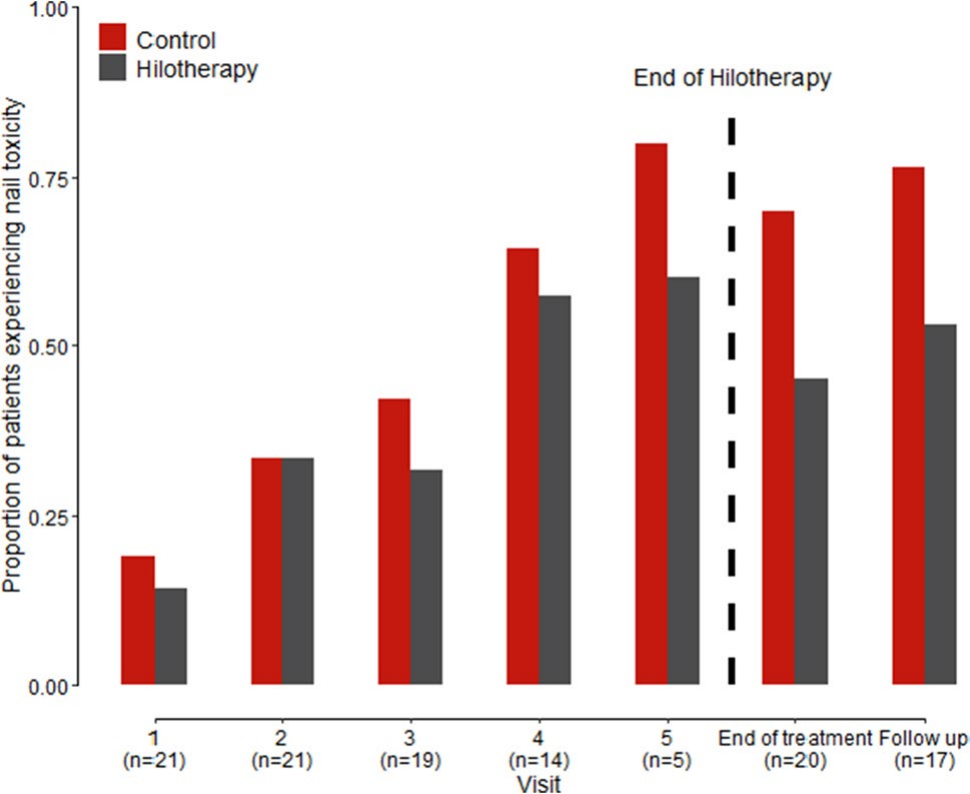
Proportion of patients experiencing pathological nail changes in the cooled vs. not cooled hand/foot by visit under Docetaxel chemotherapy (ITT, *N* = 21)

The analysis by visit for the ITT population revealed significant differences of nail toxicity across visits. More specifically, the overall risk of nail toxicity increased through visits and was lower in the cooled extremities (OR 0.42; 95% CI, 0.18–0.98; *p*-value = 0.045). This result is shown in Fig. 1.

The PPP did not show significant differences of nail toxicity in both extremities in the per visit analysis but the analysis is limited by the small patient number (online resource 6).

Nail toxicity on Paclitaxel treatment irrespective of cooling occurred in 36 patients in the ITT (78.3%; 95% CI, 63.6–89.1%) and 30 patients in the PPP (88.2%; 95% CI, 72.5–96.7%).

### Chemotherapy-induced polyneuropathy (CIPN) with Paclitaxel

A total of 34 (74%, ITT) and 28 (82%, PPP) patients receiving Paclitaxel experienced polyneuropathy at some time point. In the ITT, CIPN was grade 1 in 50%, grade 2 in 25% and grade 3 in 8% of patients (online resource 7) regardless of cooling. There were non-significant differences in the occurrence of CIPN between cooled (60.9%; 95% CI, 45.4–74.9%) vs. control (71.7%; 95% CI, 56.5–84.0%) extremities (OR: 0.61; 95% CI, 0.26–1.47; McNemar’s *p*-value = 0.059) in the ITT group. In contrast, in the PPP, a significant difference of cooled (67.6%; 95% CI, 49.5–82.6%) vs. control (82.4%; 95% CI, 65.5–93.2%) extremities (OR 0.45; 95% CI, 0.14–1.40; McNemar’s *p*-value = 0.025) was shown. Results are shown in Table 2. Looking at the severity of CIPN between the treatment arms, a numerical difference can be seen in the higher grades, where more of the non-cooled extremities suffered from higher grades compared to the cooled extremities. 16.7% of cooled extremities experienced CIPN grade II (vs. 25% patients of not-cooled extremities) and 4.2% grade III (vs. 8.3% respectively) (online resource 8).

**Table 2:** Occurrence of CIPN on Paclitaxel in any extremity at any time point (PPP, *N* = 34)

	CIPN on cooled hand/foot	
CIPN on not cooled hand/foot	No	Yes
No	6	0
Yes	5	23
McNemar’s test *p*-value = *0.025*		

In the PPP, the occurrence of grade 1 CIPN was 50%, grade 2 26.5% and grade 3 5.9% regardless of cooling. None of the patients experienced CIPN grade 4. Analysing feet and hands separately, there was a numerical difference in occurrence of CIPN in the hands in the ITT population between cooled vs. control (50.0% vs. 60.9%, McNemar’s *p*-value = 0.059) and a significant difference between cooled (52.9%) vs. control (67.6%) in the PPP (McNemar’s *p*-value = 0.025). In the feet, no statistically significant difference between cooled and control was found (58.7% vs. 63% and 64.7% vs. 73.5% for ITT and PPP, respectively).

**Fig. 2:**
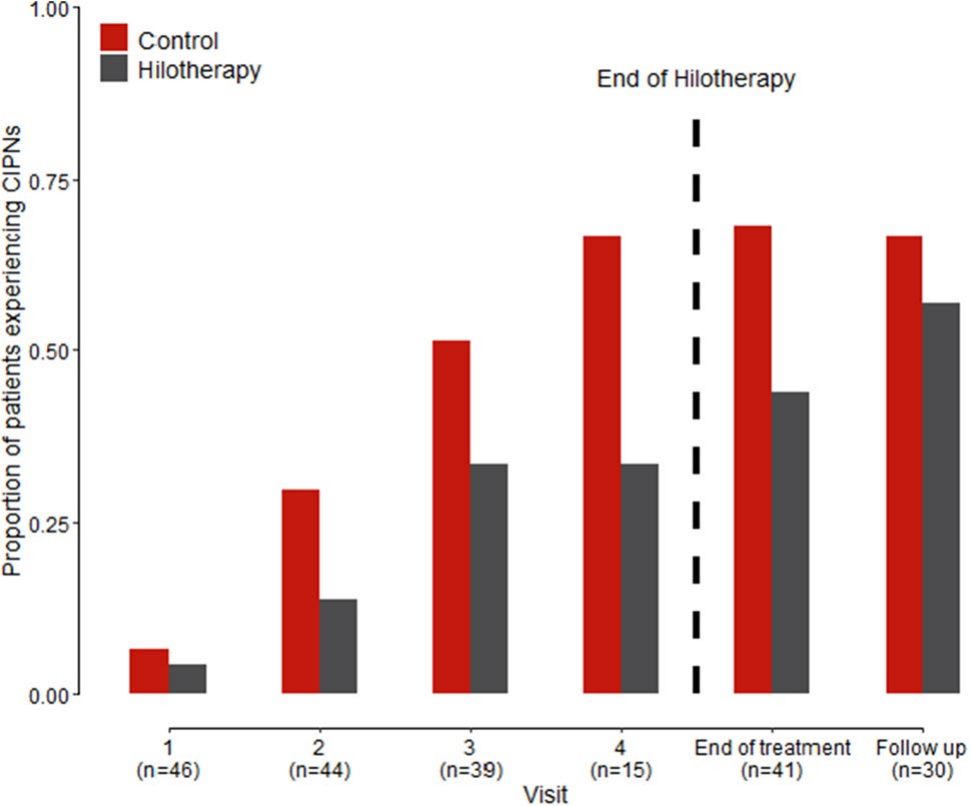
Proportion of patients experiencing CIPNs in any extremity in the cooled vs. not cooled hand/foot by visit under Paclitaxel chemotherapy (ITT, *N* = 46)

The analysis by visit revealed significant differences for the occurrence of CIPN across visits in the ITT (Table 3, Fig. 2) as well as the PPP (online resource 9 and 10).

**Table 3:** Results of the logistic mixed model for the occurrence of CIPNs in any extremity under Paclitaxel chemotherapy (ITT, *N* = 46)

Variable	*Χ*^2^	DF	*p*-value
Hilo-therapy (cooled vs. not cooled)	13.38	1	** < 0.001**
Visit	58.48	5	** < 0.001**
Hilo-therapy (cooled vs. not cooled) × visit	2.57	5	0.766

The overall risk of developing CIPN increased through visits and was significantly lower for the cooled versus control extremities (odds ratio from logistic mixed model 0.28, 95% CI 0.13–0.57, *p* = 0.001 for ITT; odds ratio 0.25, 95% CI 0.12–0.55, *p* = 0.001 for PPP). On Docetaxel treatment, 14 patients in the ITT (66.7%, 95% CI 43.0–85.4%) and 6 patients in the PPP (66.7%, 95% CI 29.9–92.5%) had occurrence of CIPN irrespective of cooling.

### Tolerance of Hilotherapy

The number of patients that experienced any adverse event due to the Hilotherapy and the number of patients that discontinued the Hilotherapy due to intolerance to the cold or patients decision are summarized in Table 4.

**Table 4:** Tolerability of the Hilotherapy for Docetaxel- and Paclitaxel-treated patients

	**Safety set (*N* = 69)**	**PPP (*N* = 43)**
**Variable**	***n* (%)**	***n* (%)**
Hilotherapy administered in control side		
No	59 (85.5%)	36 (83.7%)
Yes	10 (14.5%)	7 (16.3%)
Did the patient experience any adverse event during Hilotherapy?		
No	66 (95.7%)	41 (95.3%)
Yes	3 (4.3%)	2 (4.7%)
Hilotherapy prematurely discontinued		
No	63 (91.3%)	41 (95.3%)
Yes	6 (8.7%)	2 (4.7%)

A total of ten patients (14.5%) requested cooling of the contralateral/control extremity due to nail changes or CIPN.

## Discussion

Chemotherapy-induced polyneuropathy and nail changes are severe side effects negatively impacting the quality of life of patients. The American Society of Clinical Oncology (ASCO) Clinical Practice Guideline Summary from 2020 does not offer any guideline in prevention of these side effects [27]. Our trial confirms the high rate of nail toxicity (90% with Docetaxel and 78% with Paclitaxel) as well as CIPN (74% with Paclitaxel and 67% with Docetaxel) in the non-cooled control extremities demonstrating the unmet need for improvement of these side effects.

Several studies have previously tried to minimize these severe and disabling side effects, using the understanding that vasoconstriction prohibits the chemotherapy agent reaching the small vessels/capillaries in hands and feet.

Scotté et al. [28, 29] demonstrated a relevant reduction in nail toxicity by wearing a frozen glove or sock during administration of Docetaxel. Other studies supported these findings and concluded that a frozen sock or glove may be a useful method to reduce local side effects [30, 31].

In the case of CIPN, several studies were not successful using different prevention strategies to reduce the incidence of taxane-induced CIPN [4, 5, 12, 18]. This included the use of frozen gloves, which did not demonstrate conclusive results [15, 32]. Ohno et al. [33] found that compression therapy using stockings and sleeves in combination with medications such as goshajinkigan (Japanese herbal medicine), mecobalamin and lafutidine resulted in a reduction in CIPN, which was confirmed by another trial using surgical gloves to prevent the chemotherapy drug reaching the periphery and as a result reducing the incidence of CIPN [4]. The implication of these studies is that vasoconstriction of the extremity remains an interesting solution to pursue.

As an alternative to compression, vasoconstriction can be achieved through cryotherapy, for which the Hilotherm ChemoCare machine was created. This device has the benefit of achieving a controlled target temperature in the extremity, making it better tolerated and allowing for a controlled application of cryotherapy, as could be shown by Shaper et al. [25]. In one study, this controlled cooling appeared more effective than the use of frozen gloves [34].

Our results are in line with these previous findings. With regard to efficacy of the cooling, we could demonstrate a lower occurrence of both nail toxicity induced by Docetaxel and CIPN induced by Paclitaxel. Additionally, a higher occurrence of worse cases of CIPN in the non-cooled extremities was found.

We hypothesise that there is a dose-dependency, since results mainly reach significance in the per protocol population (with higher doses of taxane applied) and in the visit-over-time analyses (with higher cumulative doses). This is in line with the results found by Shaper et al. [25]. Looking at the per protocol population for Paclitaxel, where all patients received a cumulative dose of at least 720 mg/m^2^, significant differences in the occurrence of CIPN were seen overall as well as in hands. Lee et al. [35] describe a dependence in the incidence of CIPN according to the dose per treatment cycle, the schedule of treatment and the duration of the infusion. Our results support this with increasing significance of the cooling effect over time. The same conclusion can be made for the occurrence of pathological nail changes, where a significant difference in occurrence of nail changes in the feet could be shown in the ITT. Moreover, we found that the majority of patients did not experience adverse events related to the cooling therapy and the discontinuation rate due to intolerance was low.

Our trial has several advantages compared to the studies discussed. In contrast to other trials that only reported on CIPN grade without any comparison [25, 26], we could demonstrate the benefit within each patient by using an intrapatient control with the contralateral extremity. Moreover, our trial not only investigated CIPN but also the impact on nail toxicity.

Despite the valuable insights provided by this study, there are limitations that have to be considered when interpreting the results. The study included a relatively small number of patients, particularly in the per protocol analysis, and was only conducted in one centre. Because of the insufficient recruitment of patients compared to the statistical assumptions, the study was overall underpowered which increases the risk of false positive results. As an open-label study, both the patients and the healthcare providers were aware of the cooling therapy being administered and the assessments were of subjective nature, potentially also leading to bias.

Additionally, there might be a confounding effect, as the dominant hand was cooled in most patients. Neuropathic symptoms such as numbness or weakness can lead to more severe impacts on activities of daily life on the dominant hand, therefore resulting in cooling having a greater effect than on the non-dominant extremity. Another confounding factor is the fact that 14.5% of patients elected to receive cooling of the control extremity in addition to the already cooled extremities. This might have led to an underestimation of the cooling effect.

Overall, the findings of this study provide additional evidence supporting the potential benefits of using the Hilotherapy machine as a preventive, well-tolerated measure for reducing nail toxicity and CIPN in patients receiving taxane chemotherapy. The Hilotherapy machine demonstrates potential to reduce nail toxicity and CIPN in patients undergoing taxane chemotherapy, particularly in cases of higher cumulative doses and prolonged treatment. Larger studies are warranted to confirm these findings and establish the efficacy of Hilotherapy in managing these chemotherapy-related adverse effects.

### Supplementary Information

Below is the link to the electronic supplementary material.

Supplementary file1 (PDF 68 KB)

Supplementary file2 (PDF 86 KB)

Supplementary file3 (PDF 75 KB)

Supplementary file4 (PDF 79 KB)

Supplementary file5 (PDF 49 KB)

Supplementary file6 (PDF 76 KB)

Supplementary file7 (PDF 55 KB)

Supplementary file8 (PDF 56 KB)

Supplementary file9 (PDF 65 KB)

Supplementary file10 (PDF 73 KB)

## Data Availability

The data that support the findings of this study are available from the corresponding author, [RC], upon reasonable request.
