# Supplementary material for: Prevention of taxane chemotherapy-induced nail changes and peripheral neuropathy by application of extremity cooling: a prospective single-centre study with intrapatient comparison
Source: Support Care Cancer. 2024 Jul 27;32(8):554. doi: 10.1007/s00520-024-08737-3 (PMC11283420; doi:10.1007/s00520-024-08737-3)
Supplement: Supplementary file 1 — Supplementary file1 (PDF 68 KB) [file 520_2024_8737_MOESM1_ESM.pdf]

Prevention of taxane chemotherapy induced nail changes and peripheral neuropathy by application of extremity cooling: a prospective single centre study with intrapatient comparison.

Supportive Care of Cancer

Kristen Johnson[1,2], Barbara Stoffel[1], Michael Schwitter[1], Stefanie Hayoz[3], Alfonso Rojas Mora[3], Angela Fischer[1], Tämer El Saadany[1], Ursula Hasler[1], Roger von Moos[1], Annalea Patzen[1], Michael Mark[2], Gillian Roberts[1], Richard Cathomas[1]

Affiliations

[1] Division of Oncology/Hematology, Kantonsspital Graubünden, Chur, Switzerland

[2] Department of Internal Medicine, Kantonsspital Graubünden, Chur, Switzerland

[3] SAKK Competence Center, Bern, Switzerland

Corresponding author

Richard Cathomas, MD

Email: richard.cathomas@ksgr.ch

Table 1 Evaluation of nail changes and allocation to CTCAE Version 5.0

	Rechte Hand	Linke Hand	Rechter Fuss	Linker Fuss
Hilotherapie:	☐ Ja ☐Nein	☐Ja ☐Nein	☐Ja ☐Nein	☐Ja ☐Nein
Nagelveränderungen				

Bitte<br>ffendes<br>ankreuzen	☐ Hyperpigmentation<br>☐ Verfärbungen<br>☐ Melanonychia<br>☐ Leukonychia<br>☐ Splinter Hämorrhagie<br>☐ Beau Lines<br>☐ Nail ridging<br>☐ Koilonychia<br>☐ Akute Paronychia<br>☐ Subunguales Hämatom<br>☐ Subunguale Hyperkeratose<br>☐ Partielle Onycholyse<br>☐ Vollständige Onycholyse<br>☐ Subungualer Abszess<br>☐ Leichte Schmerzen<br>☐ Starke Schmerzen<br>☐ Sonstiges	☐ Hyperpigmentation<br>☐ Verfärbungen<br>☐ Melanonychia<br>☐ Leukonychia<br>☐ Splinter Hämorrhagie<br>☐ Beau Lines<br>☐ Nail ridging<br>☐ Koilonychia<br>☐ Akute Paronychia<br>☐ Subunguales Hämatom<br>☐ Subunguale Hyperkeratose<br>☐ Partielle Onycholyse<br>☐ Vollständige Onycholyse<br>☐ Subungualer Abszess<br>☐ Leichte Schmerzen<br>☐ Starke Schmerzen<br>☐ Sonstiges	☐ Hyperpigmentation<br>☐ Verfärbungen<br>☐ Melanonychia<br>☐ Leukonychia<br>☐ Splinter Hämorrhagie<br>☐ Beau Lines<br>☐ Nail ridging<br>☐ Koilonychia<br>☐ Akute Paronychia<br>☐ Subunguales Hämatom<br>☐ Subunguale Hyperkeratose<br>☐ Partielle Onycholyse<br>☐ Vollständige Onycholyse<br>☐ Subungualer Abszess<br>☐ Leichte Schmerzen<br>☐ Starke Schmerzen<br>☐ Sonstiges	☐ Hyperpigmentation<br>☐ Verfärbungen<br>☐ Melanonychia<br>☐ Leukonychia<br>☐ Splinter Hämorrhagie<br>☐ Beau Lines<br>☐ Nail ridging<br>☐ Koilonychia<br>☐ Akute Paronychia<br>☐ Subunguales Hämatom<br>☐ Subunguale Hyperkeratose<br>☐ Partielle Onycholyse<br>☐ Vollständige Onycholyse<br>☐ Subungualer Abszess<br>☐ Leichte Schmerzen<br>☐ Starke Schmerzen<br>☐ Sonstiges
**Nagelhärter angewendet:**	☐Ja ☐Nein	☐Ja ☐Nein	☐Ja ☐Nein	☐Ja ☐Nein

**Grad I**<br>Bitte zutreffendes<br>ankreuzen	☐ Hyperpigmentation<br>☐ Verfärbungen<br>☐ Melanonychia<br>☐ Leukonychia<br>☐ Splinter haemorrhage<br>☐ Beau Lines<br>☐ Nail ridging<br>☐ Koilonychia<br>☐ Leichte Schmerzen
**Grad II**<br>Bitte zutreffendes<br>ankreuzen	☐ Akute Paronychia<br>☐ Subunguales Hämatom<br>☐ Subunguale Hyperkeratose<br>☐ Partielle Onycholyse

	☐ Vollständige Onycholyse<br>☐ Subungualer Abszess<br>☐ Starke Schmerzen
