# Supplementary material for: Prevention of taxane chemotherapy-induced nail changes and peripheral neuropathy by application of extremity cooling: a prospective single-centre study with intrapatient comparison
Source: Support Care Cancer. 2024 Jul 27;32(8):554. doi: 10.1007/s00520-024-08737-3 (PMC11283420; doi:10.1007/s00520-024-08737-3)
Supplement: Supplementary file 2 — Supplementary file2 (PDF 86 KB) [file 520_2024_8737_MOESM2_ESM.pdf]

Prevention of taxane chemotherapy induced nail changes and peripheral neuropathy by application of extremity cooling: a prospective single centre study with intrapatient comparison.

Supportive Care of Cancer

Kristen Johnson[1,2], Barbara Stoffel[1], Michael Schwitter[1], Stefanie Hayoz[3], Alfonso Rojas Mora[3], Angela Fischer[1], Tämer El Saadany[1], Ursula Hasler[1], Roger von Moos[1], Annalea Patzen[1], Michael Mark[2], Gillian Roberts[1], Richard Cathomas[1]

Affiliations

[1] Division of Oncology/Hematology, Kantonsspital Graubünden, Chur, Switzerland

[2] Department of Internal Medicine, Kantonsspital Graubünden, Chur, Switzerland

[3] SAKK Competence Center, Bern, Switzerland

Corresponding author

Richard Cathomas, MD

Email: richard.cathomas@ksgr.ch

Table 2 PNQ-Questionnaire

	**Rechte Hand**	**Linke Hand**	**Rechter Fuss**	**Linker Fuss**
Hilotherapie:	☐ Ja<br>☐ Nein	☐ Ja<br>☐ Nein	☐ Ja<br>☐ Nein	☐ Ja<br>☐ Nein
**PNQ <u>Sensorisch</u>** *Bitte zutreffendes Feld ankreuzen (pro Hand bzw. Fuss ein Kreuz)*				
**<u>Grad A</u>**<br>Ich spüre kein Taubheitsgefühl, Kribbeln oder Prickeln				
**<u>Grad B</u>**<br>Ich spüre leichtes Taubheitsgefühl, Kribbeln oder Prickeln. Diese führen zu keinen Beeinträchtigungen im Alltag.				
**<u>Grad C</u>**<br>Ich spüre mittelschweres Taubheitsgefühl, Kribbeln oder Prickeln. Diese führen zu keinen Beeinträchtigungen im Alltag.				
**<u>Grad D*</u>**<br>Ich spüre mittelschweres bis schweres Taubheitsgefühl, Kribbeln oder Prickeln. Diese führen zu Beeinträchtigungen im Alltag.				
**<u>Grad E*</u>**<br>Ich spüre schweres Taubheitsgefühl, Kribbeln oder Prickeln. Diese hindern mich beim Verrichten der täglichen Aufgaben				

* Falls D oder E zutrifft, bitte unten stehende Tabelle **<u>auch</u>** ausfüllen

	**Rechte Hand**	**Linke Hand**	**Rechter Fuss**	**Linker Fuss**
Hilotherapie:	☐ Ja<br>☐ Nein	☐ Ja<br>☐ Nein	☐ Ja<br>☐ Nein	☐ Ja<br>☐ Nein
**PNQ <u>Motorisch</u>** *Bitte zutreffendes Feld ankreuzen (pro Hand bzw. Fuss ein Kreuz)*				
**<u>Grad A</u>**<br>Ich spüre kein Schwächegefühl				
**<u>Grad B</u>**<br>Ich spüre eine leichte Schwäche. Diese führt zu keinen Beeinträchtigungen im Alltag.				
**<u>Grad C</u>**<br>Ich spüre eine mittelschwere Schwäche. Diese führt zu keinen Beeinträchtigungen im Alltag.				
**<u>Grad D*</u>**<br>Ich spüre eine mittelschwere bis schwere Schwäche. Diese führt zu Beeinträchtigungen im Alltag.				
**<u>Grad E*</u>**<br>Ich spüre eine schwere Schwäche. Diese hindert mich am Verrichten der täglichen Aufgaben				

* Falls D oder E zutrifft, bitte unten stehende Tabelle **<u>auch</u>** ausfüllen

---

Bitte markieren Sie mit einem X zutreffende Kästchen:

Ich verspüre Einschränkungen bei folgenden Aufgaben:

☐ Knöpfe zuknöpfen<br>☐ Messer verwenden<br>☐ Gabel verwenden<br>☐ Löffel verwenden<br>☐ Andere Essutensilien	☐ Türe öffnen<br>☐ Kontaktlinsen Gebrauch<br>☐ Telefon Gebrauch<br>☐ Fernbedienung Gebrauch<br>☐ Tastatur benützen	☐ Schmuck anziehen<br>☐ Nähen<br>☐ Stricken<br>☐ Schuhe binden	☐ Schlafen<br>☐ Treppen steigen<br>☐ Schreiben<br>☐ Arbeiten<br>☐ Laufen<br>☐ Fahren

☐ Andere:

____
