# Supplementary material for: Prevention of taxane chemotherapy-induced nail changes and peripheral neuropathy by application of extremity cooling: a prospective single-centre study with intrapatient comparison
Source: Support Care Cancer. 2024 Jul 27;32(8):554. doi: 10.1007/s00520-024-08737-3 (PMC11283420; doi:10.1007/s00520-024-08737-3)
Supplement: Supplementary file 3 — Supplementary file3 (PDF 75 KB) [file 520_2024_8737_MOESM3_ESM.pdf]

Prevention of taxane chemotherapy induced nail changes and peripheral neuropathy by application of extremity cooling: a prospective single centre study with intrapatient comparison.

Supportive Care of Cancer

Kristen Johnson[1,2], Barbara Stoffel[1], Michael Schwitter[1], Stefanie Hayoz[3], Alfonso Rojas Mora[3], Angela Fischer[1], Tämer El Saadany[1], Ursula Hasler[1], Roger von Moos[1], Annalea Patzen[1], Michael Mark[2], Gillian Roberts[1], Richard Cathomas[1]

Affiliations

[1] Division of Oncology/Hematology, Kantonsspital Graubünden, Chur, Switzerland

[2] Department of Internal Medicine, Kantonsspital Graubünden, Chur, Switzerland

[3] SAKK Competence Center, Bern, Switzerland

Corresponding author

Richard Cathomas, MD

Email: richard.cathomas@ksgr.ch

Table 3 CTCAE Version 5.0 for evaluation of CIPN

	Rechte Hand	Linke Hand	Rechter Fuss	Linker Fuss
Hilotherapie:	☐ Ja<br>☐ Nein	☐ Ja<br>☐ Nein	☐ Ja<br>☐ Nein	☐ Ja<br>☐ Nein
**CTCAE Version 5.0: Sensorisch**				
**Grad 0**<br>Asymptomatic<br>(keine Symptome)				
**Grad I**<br>Paraesthesia<br>(Parästhesie ohne weitere Symptome)				
**Grad II**<br>Moderate symptoms; limiting instrumental ADL[1]<br>(moderate Symptome mit Einschränkungen im Alltag)				
**Grad III**<br>Severe symptoms; limiting self care ADL<br>(schwere Symptome mit Einschränkung in der Eigenversorgung im Alltag)				
**Grad IV**<br>Life-threatening consequences; urgent intervention indicated<br>(lebensbedrohliche Konsequenzen, sofortige Interventionen nötig)				

[1] ADL = Activities of daily living (Aktivitäten im täglichen Leben)

	**Rechte Hand**	**Linke Hand**	**Rechter Fuss**	**Linker Fuss**
Hilotherapie:	☐ Ja<br>☐ Nein	☐ Ja<br>☐ Nein	☐ Ja<br>☐ Nein	☐ Ja<br>☐ Nein
**CTCAE Version 5.0: <u>Motorisch</u>**				
**<u>Grad 0</u>**<br>Asymptomatic<br>(keine Symptome)				
**<u>Grad I</u>**<br>Mild symptoms; clinical or diagnostic observation only<br>(milde Symptome, nur Observation)				
**<u>Grad II</u>**<br>Moderate symptoms; limiting instrumental ADL<br>(moderate Symptome mit Einschränkungen im Alltag)				
**<u>Grad III</u>**<br>Severe symptoms; limiting self care ADL<br>(schwere Symptome mit Einschränkung in der Eigenversorgung im Alltag)				
**<u>Grad IV</u>**<br>Life-threatening consequences; urgent intervention indicated<br>(lebensbedrohliche Konsequenzen, sofortige Interventionen nötig)				
