# Supplementary material for: Prevention of taxane chemotherapy-induced nail changes and peripheral neuropathy by application of extremity cooling: a prospective single-centre study with intrapatient comparison
Source: Support Care Cancer. 2024 Jul 27;32(8):554. doi: 10.1007/s00520-024-08737-3 (PMC11283420; doi:10.1007/s00520-024-08737-3)
Supplement: Supplementary file 4 — Supplementary file4 (PDF 79 KB) [file 520_2024_8737_MOESM4_ESM.pdf]

Prevention of taxane chemotherapy induced nail changes and peripheral neuropathy by application of extremity cooling: a prospective single centre study with intrapatient comparison.

Supportive Care of Cancer

Kristen Johnson[1,2], Barbara Stoffel[1], Michael Schwitter[1], Stefanie Hayoz[3], Alfonso Rojas Mora[3], Angela Fischer[1], Tämer El Saadany[1], Ursula Hasler[1], Roger von Moos[1], Annalea Patzen[1], Michael Mark[2], Gillian Roberts[1], Richard Cathomas[1]

Affiliations

[1] Division of Oncology/Hematology, Kantonsspital Graubünden, Chur, Switzerland

[2] Department of Internal Medicine, Kantonsspital Graubünden, Chur, Switzerland

[3] SAKK Competence Center, Bern, Switzerland

Corresponding author

Richard Cathomas, MD

Email: richard.cathomas@ksgr.ch

Table 4 Schedule of assessments for weekly application[1]

Assessment	Baseline assessment	Cycle Visit			End of treatment	Final assessment
**Timeline**	-28d to -1d	day 21	day 49	day 77	14d – 28d after last treatment	42d – 53d after last treatment
**Patient information and written consent**	x					
**Inclusion-/ Exclusion criteria**	x					
**Medical history**	x					
**Participant characteristics**	x					
**Hilo-therapy**	x	x	x	x		
**CTCAE Questionnaire**	x	x	x	x	x	x
**PNQ Questionnaire**	x	x	x	x	x	x
**Nail assessment**	x	x	x	x	x	x
**Photo Documentation**	x				x	x

Table 5 Schedule of assessments for bi-weekly application[2]

Assessment	Baseline assessment	Cycle Visit				End of treatment	Final assessment
**Timeline**	-28d to -1d	day 28	day 56	day 84	day 110	14d – 28d after last treatment	42d – 53d after last treatment
**Patient information and written consent**	x						
**Inclusion-/ Exclusion criteria**	x						
**Medical history**	x						

[1] Assessment on the 4th, 8th and 12th cycle
[2] Assessment on every second cycle

**Participant characteristics**	x						
**Hilo-therapy**	x	x	x	x	x		
**CTCAE Questionnaire**	x	x	x	x	x	x	x
**PNQ Questionnaire**	x	x	x	x	x	x	x
**Nail assessment**	x	x	x	x	x	x	x
**Photo Documentation**	x					x	x

Table 6 Schedule of assessments for application every 3 weeks[3]

**Assessment**	**Baseline assessment**	**Cycle Visit**						**End of treatment**	**Final assessment**
**Timeline**	-28d to -1d	day 21	day 42	day 63	day 84	day 105	day 126	14d – 28d after last treatment	42d – 53d after last treatment
**Patient information and written consent**	x								
**Inclusion-/ Exclusion criteria**	x								
**Medical history**	x								
**Participant characteristics**	x								
**Hilo-therapy**	x	x	x	x	x	x	x		
**CTCAE Questionnaire**	x	x	x	x	x	x	x	x	x
**PNQ Questionnaire**	x	x	x	x	x	x	x	x	x
**Nail assessment**	x	x	x	x	x	x	x	x	x
**Photo Documentation**	x							x	x

[3] Assessment on every cycle
