# Supplementary material for: Prevention of taxane chemotherapy-induced nail changes and peripheral neuropathy by application of extremity cooling: a prospective single-centre study with intrapatient comparison
Source: Support Care Cancer. 2024 Jul 27;32(8):554. doi: 10.1007/s00520-024-08737-3 (PMC11283420; doi:10.1007/s00520-024-08737-3)
Supplement: Supplementary file 5 — Supplementary file5 (PDF 49 KB) [file 520_2024_8737_MOESM5_ESM.pdf]

Prevention of taxane chemotherapy induced nail changes and peripheral neuropathy by application of extremity cooling: a prospective single centre study with intrapatient comparison.

Supportive Care of Cancer

Kristen Johnson[1,2], Barbara Stoffel[1], Michael Schwitter[1], Stefanie Hayoz[3], Alfonso Rojas Mora[3], Angela Fischer[1], Tämer El Saadany[1], Ursula Hasler[1], Roger von Moos[1], Annalea Patzen[1], Michael Mark[2], Gillian Roberts[1], Richard Cathomas[1]

Affiliations

[1] Division of Oncology/Hematology, Kantonsspital Graubünden, Chur, Switzerland

[2] Department of Internal Medicine, Kantonsspital Graubünden, Chur, Switzerland

[3] SAKK Competence Center, Bern, Switzerland

Corresponding author

Richard Cathomas, MD

Email: richard.cathomas@ksgr.ch

Table 5 Reasons for dose modification

Variable	Total (N=48) n (%)
Reason for Paclitaxel dose reduction	
. Deterioration of general condition	12 (25%)
. Myelosuppression	5 (10.4%)
. Progressive CIPN	3 (6.3%)
. Refractory Nausea	2 (4.1%)
Reason for Docetaxel dose reduction	**(N=21)**
. Deterioration of general	5 (23.8%)
