# Supplementary material for: Prevention of taxane chemotherapy-induced nail changes and peripheral neuropathy by application of extremity cooling: a prospective single-centre study with intrapatient comparison
Source: Support Care Cancer. 2024 Jul 27;32(8):554. doi: 10.1007/s00520-024-08737-3 (PMC11283420; doi:10.1007/s00520-024-08737-3)
Supplement: Supplementary file 7 — Supplementary file7 (PDF 55 KB) [file 520_2024_8737_MOESM7_ESM.pdf]

Prevention of taxane chemotherapy induced nail changes and peripheral neuropathy by application of extremity cooling: a prospective single centre study with intrapatient comparison.

Supportive Care of Cancer

Kristen Johnson[1,2], Barbara Stoffel[1], Michael Schwitter[1], Stefanie Hayoz[3], Alfonso Rojas Mora[3], Angela Fischer[1], Tämer El Saadany[1], Ursula Hasler[1], Roger von Moos[1], Annalea Patzen[1], Michael Mark[2], Gillian Roberts[1], Richard Cathomas[1]

Affiliations

[1] Division of Oncology/Hematology, Kantonsspital Graubünden, Chur, Switzerland

[2] Department of Internal Medicine, Kantonsspital Graubünden, Chur, Switzerland

[3] SAKK Competence Center, Bern, Switzerland

Corresponding author

Richard Cathomas, MD

Email: richard.cathomas@ksgr.ch

Table 7 Polyneuropathies during Paclitaxel administration and up to 56 days after the end of treatment by max. grade for the safety set (N=48).

Max. grade	PN CTCAE - Motor	PN CTCAE - Sensory	Any PN CTCAE
Grade 0	17 (35.4%)	10 (20.8%)	8 (16.7%)
Grade 1	19 (39.6%)	24 (50.0%)	24 (50.0%)
Grade 2	10 (20.8%)	10 (20.8%)	12 (25.0%)
Grade 3	2 (4.2%)	4 (8.3%)	4 (8.3%)
Grade 4	0 (0.0%)	0 (0.0%)	0 (0.0%)
