# Supplementary material for: Prevention of taxane chemotherapy-induced nail changes and peripheral neuropathy by application of extremity cooling: a prospective single-centre study with intrapatient comparison
Source: Support Care Cancer. 2024 Jul 27;32(8):554. doi: 10.1007/s00520-024-08737-3 (PMC11283420; doi:10.1007/s00520-024-08737-3)
Supplement: Supplementary file 8 — Supplementary file8 (PDF 56 KB) [file 520_2024_8737_MOESM8_ESM.pdf]

Prevention of taxane chemotherapy induced nail changes and peripheral neuropathy by application of extremity cooling: a prospective single centre study with intrapatient comparison.

Supportive Care of Cancer

Kristen Johnson[1,2], Barbara Stoffel[1], Michael Schwitter[1], Stefanie Hayoz[3], Alfonso Rojas Mora[3], Angela Fischer[1], Tämer El Saadany[1], Ursula Hasler[1], Roger von Moos[1], Annalea Patzen[1], Michael Mark[2], Gillian Roberts[1], Richard Cathomas[1]

Affiliations

[1] Division of Oncology/Hematology, Kantonsspital Graubünden, Chur, Switzerland

[2] Department of Internal Medicine, Kantonsspital Graubünden, Chur, Switzerland

[3] SAKK Competence Center, Bern, Switzerland

Corresponding author

Richard Cathomas, MD

Email: richard.cathomas@ksgr.ch

Table 8 Severity of CIPN between the treatment arms in Patients receiving Paclitaxel

Variable	Control (N=48) n (%)	Hilotherapy (N=48) n (%)
Max. grade		
. Grade 0	9 (18.8%)	13 (27.1%)
. Grade 1	23 (47.9%)	25 (52.1%)
. Grade 2	12 (25.0%)	8 (16.7%)
. Grade 3	4 (8.3%)	2 (4.2%)
. Grade 4	0 (0.0%)	0 (0.0%)
